# Transition Metal (Fe, Co, Ni) Nanoparticles on Selective Amino-N-Doped Carbon as High-Performance Oxygen Reduction Reaction Electrocatalyst

**DOI:** 10.3390/nano9050742

**Published:** 2019-05-14

**Authors:** Seonghee Kim, Shuhei Kato, Takahiro Ishizaki, Oi Lun Li, Jun Kang

**Affiliations:** 1 School of Materials Science and Engineering, Pusan National University, Busan 46241, Korea; ksh08280@naver.com; 2 Department of Materials Science and Engineering, College of Engineering, Shibaura Institute of Technology, Tokyo 135-8548, Japan; mb18008@shibaura-it.ac.jp (S.K.); ishizaki@shibaura-it.ac.jp (T.I.); 3 Division of Marine Engineering, Korea Maritime and Ocean University, Busan 606-791, Korea

**Keywords:** nitrogen-doped carbon, transitional metal nanoparticles, oxygen reduction reaction, metal-nitrogen-hybrid catalyst, selective amino-N doping

## Abstract

Metal-air batteries are attracting increasing attention as a superior renewable energy conversion device due to their high performance and strong potential. However, the high cost and low stability of the current Pt catalyst is the main obstacle preventing wide industrial application. In this work, we applied a plasma process to fabricate aniline and a transition metals electrode (Fe, Co, Ni) as the carbon-nitrogen and the metal nanoparticle (NP) precursors, respectively, for selective metal/amino-N-doped carbon catalysts. All three as-synthesized catalysts exhibited dominant amino-N as the major C–N bonding state. In electrochemical testing, Co/amino-N-doped carbon showed positive ***E*_1/2_** potential (0.83 V vs. Reversible Hydrogen Electrode (RHE)). In addition, the calculated electron transfer number (*n*) of Co/amino-N-doped carbon at 0.5 V vs. RHE was 3.81, which was only slightly less than that of commercial Pt/C (3.97). This superior performance of transition metal/amino-N-doped carbon promotes it as an economical oxygen reduction reaction (ORR) electrocatalyst to replace expensive Pt/C in metal-air batteries.

## 1. Introduction

Within the accelerating application of renewable energy in many fields, metal-air batteries have been spotlighted as one of the candidates for a next-generation energy conversion device, mainly due to their high theoretical energy density, simple structure, and variety of component designs [1,2,3,4]. When air is used as the cathode fuel, the oxygen reduction reaction (ORR) that occurs at the cathode governs the performance of the overall system. However, due to the difficulties of O_2_ chemical sorption/activation, the slow reaction rate of ORR often limits the system performance [4,5]. To date, platinum (Pt) shows the best performance as an ORR catalyst and has often been applied in practical metal-air batteries. Commercial Pt catalysts are composed of 20 wt.% Pt nanoparticles (NPs) on a high-surface-area carbon support. Nevertheless, the high capital cost and scarcity of Pt have prompted researchers to develop other alternatives to replace Pt/C [6].

Recently, nitrogen-doped carbon (N-doped carbon), which is a sub-group of the heteroatom-doped carbon catalysts, has attracted much attention due to its high catalytic performance and superior stability [7,8,9,10,11,12,13]. The catalyst often shows relatively high catalytic performance in alkaline electrolytes. In the case of N-doped carbon, nitrogen reduces the charge density of the nearby carbon atoms due to the differences in electronegativity of carbon and nitrogen. As a result, the nitrogen dopant can induce charge delocalization, which not only polarizes carbon to a positive charge to promote adsorption of the O_2_ molecule but also promotes ORR by changing the chemisorption mode of O_2_ from end-on adsorption (Pauling model) to side-on adsorption (Yeager model) [7,14,15]. Many studies suggest that total N content does not contribute significantly to improving ORR, whereas the bonding state of the N atom does [16]. The improvement in ORR activity has been attributed to pyridinic-N and graphitic-N by many studies [17,18,19,20]. Also, selective doping of graphitic-N and pyridinic-N can be easily controlled by heat treatment due to their high thermal stability, and different C–N bonding states affect the different performances of ORR, such as the selective electron pathway (4e^−^ or 2e^−^), current density and onset potential [17]. By using this concept, the performance can be improved by tuning towards dominant pyridinic-N bonding or by controlling the ratio of pyridinic-N/pyrrolic-N for the selective 2e^−^ pathway in industrial H_2_O_2_ synthesis [21,22]. Compared to metal-free N-doped carbon catalysts, the doping of transition metals such as Fe, Ni, and Co on N-doped carbon support further enhanced the ORR catalytic activities. Many studies proposed that the metal dopant (especially Fe, Co) on N-doped carbon coordinated by nitrogen groups, such as pyridinic-N or metal-N moieties (FeN_2_, FeN_4_, CoN_4_, CoN_2_), enhances the ORR activity [23,24,25,26,27,28,29,30]. For instance, Kramm et al. reported Fe–N moieties (FeN_4_) can improve ORR activity significantly without the presence of inorganic metal nanoparticles [27]. Peng et al. reported a high catalytic performance of Fe–N-doped carbon with dominant graphitic-N and pyridinic-N in both alkaline and acidic electrolytes [29]. Du et al. fabricated various types of transition metal (Fe, Ni, Co) catalyst on N-doped carbon, and reported that Fe–N-doped carbon showed the highest potential of 0.96 V [30]. Other researches also showed that not only M-N_x_ moieties, but also metal nanoparticles (NPs) can improve ORR performance. Peng et al. reported superior ORR onset potential for Fe and Co on N-doped carbon derived from polyaniline and melamine, and suggested that the transition metals (Fe and Co) provided a synergic effect between the metal NPs and N-doped carbon matrix [31]. Tang et al. synthesized porous nitrogen-doped graphitic carbon fibers embedded with metal and metal oxide particles. In particular, Co, Co_3_O_4_ with N-doped carbon support exhibited comparable ORR performance to Pt/C in alkaline media with high stability and strong methanol tolerance [32]. Zeng et al. reported Fe–N-doped carbon composed with FeN particles covered by graphitic carbon layers with a high specific surface area. The as-synthesized catalysts showed superior ORR performance, including high ORR onset potential and low peroxide yield rate, which were both comparable to that of Pt/C [33].

A few studies also suggested that amino-N could enhance ORR activity, especially in the onset potential of reaction. Due to the electron donating effect of an amine group, amino-N works as an electron donating group in N-doped carbon and improves the electron conductivity of carbon support [34,35,36]. However, amino-N has the lowest thermal instability (<550 °C) among all C–N chemical bonds [34]. Thus, it is very challenging to dope amino-N selectively by conventional high-temperature chemical vapor deposition or post-annealing treatment. Zhang et al. first fabricated selective amino-N-doped carbon catalysts by the hydrothermal method at a moderate temperature of 200 °C. Their study showed that amino-N enhances ORR activity with a more positive onset potential [34]. Li et al. applied a room-temperature plasma process to synthesize selective amino-N and pyridinic-N by varying the liquid precursors. The ORR onset potential was enhanced with a higher ratio of amino-N [36]. To date, the synergic effect of transition metals on amino-N-doped carbon has yet to be reported.

As the plasma process is a room-temperature bottom-up method, it is beneficial for maintaining the molecular structure, in particular, amino-N with the lowest thermal stability among all types of C–N bonding. In addition, transition metal NPs can be generated simultaneously by electrode sputtering during plasma discharge [37,38,39,40,41,42,43,44]. In this study, we applied the same process to fabricate transition metal (Fe, Ni or Co) NPs on selective amino-N-doped carbon catalysts. Aniline was chosen as a precursor for dominant amino-N bonding, while the pure metal electrodes were the precursor of Fe, Co or Ni NPs. The physical and chemical properties of three electrocatalysts were firstly characterized, and their corresponding electrochemical performance was evaluated in detail and compared to that of commercial 20 wt.% Pt/C.

## 2. Materials and Methods

### 2.1. Synthesis of Metal (Fe, Co, Ni)-N/C Catalyst by A Plasma Process

**Figure 1:**
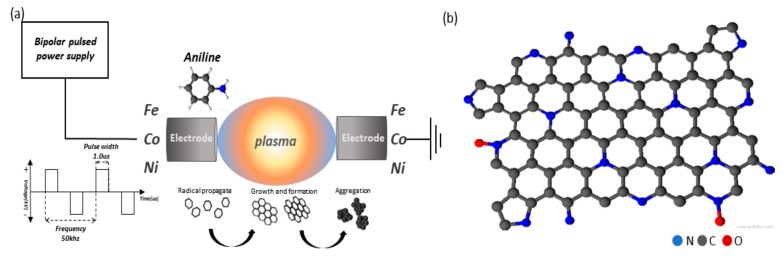
(**a**) Schematic illustration of the solution plasma process and synthesis of metal (Fe, Co, Ni)-N/C catalyst, and (**b**) structure of N-doped carbon catalyst.

The plasma was discharged between a pair of high purity transition metal (Fe, Co, Ni) electrodes (99.999%, Nilaco Co., Ltd., Tokyo, Japan, diameter of 1 mm) inside a pure aniline solution (purity >99%, Junsei Chemical Co., Ltd., Tokyo, Japan) by using a bipolar pulse power supply (MPP-HV02, kurita Seisakusho, Kyoto, Japan). The plasma was discharged at a voltage of ~4 kV, a frequency of 50 kHz, and a pulse width of 1.0 μs (Figure 1a). A stable plasma was discharged for 30 min to obtain ~1 g samples. The solution was filtered using a Φ 55 mm polytetrafluoroethylene filter, and the resulting filtered carbon powder samples were dried in an oven for 10 h at 80 °C. Finally, the samples were heated to 800 °C for 1 h under nitrogen gas in order to improve their electrical conductivity. We hereafter named the as-synthesized samples as Fe-N/C, Co-N/C, and Ni-N/C. The schematical structure of N-doped carbon support is shown in Figure 1b.

### 2.2. Structure and Chemical Composition Analysis

The nitrogen absorption–desorption method (BET, Brunauer Emmett Teller; TriStar-II3020, Shimadzu, Tokyo, Japan,) was used for analyzing the surface area, pore volume and pore diameter of the synthesized metal-N/C powder. For morphology and chemical composition analysis, the samples were characterized by scanning electron microscopy (SEM; JEOL, JSM-7100F, Tokyo, Japan), X-ray diffraction (XRD; Rigaku, Ultima IV, Tokyo, Japan) and X-ray photoelectron spectroscopy (XPS; JEOL, JPS-9010MC, Tokyo, Kanto, Japan).

### 2.3. Electrochemical Measurements

An electrochemical analyzer (Biologic, VSP, Grenoble, France) was used to analyze the electrochemical properties of the synthesized N-doped carbon samples. The catalyst ink for the electrochemical analysis was made by adding 5 mg of well ground metal (Fe, Ni, Co)-N/C catalyst into a mixture composed of 280 μL of distilled water, 200 μL of ethanol and 20 μL of Nafion®117 Solution, which was then ultrasonicated for 30 min. A total of 5 μL of well-dispersed catalyst ink was applied onto a well-polished GC disk (diameter: 3 mm) electrode (working electrode), where a platinum coil and Ag/AgCl (saturated KCl) were used as the counter and reference electrodes, respectively. After the three-electrode cell was prepared, cyclic voltammetry (CV) was measured in O_2_ saturated 0.1M KOH with a scan rate of 50 mVs^−1^ and a potential range of −1.0 V to 0.4 V. Linear sweep voltammetry (LSV) was conducted in O_2_ saturated 0.1M KOH with a scan rate of 10 mVs^−1^ and rotating speed of 1600 rpm, between a potential range of 0 to 1.2 V vs. RHE.

## 3. Results

### 3.1. Properties of Metal (Fe, Ni, Co)-N/C Catalyst

**Figure 2:**
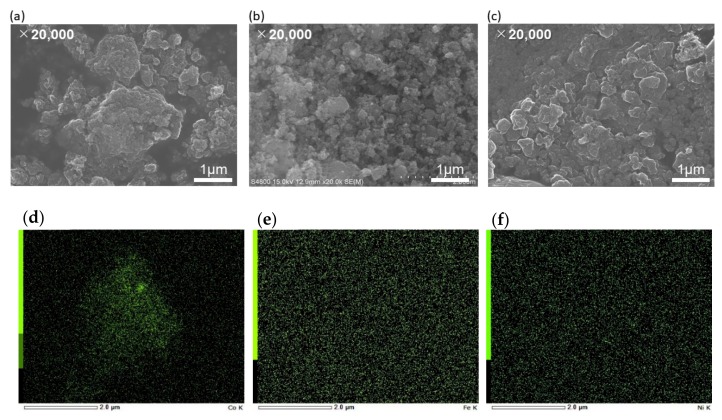
Scanning Electron Microscopy (SEM) images of (**a**,**d**) Co-N/C, (**b**,**e**) Fe-N/C, and (**c**,**f**) Ni-N/C at magnification of ×20,000 and their corresponding EDS images of metal NPs, respectively.

The SEM images reveal the morphology of metal (Co, Fe, Ni)-N/C in Figure 2a–c. During the plasma discharge, the resulting radicals initially formed short graphitic-like layers that finally aggregated into sphere-like structures. The structure of the N-doped carbon catalysts consists of interconnected nanocarbon balls. From Table 1, certain amount of nitrogen in the aniline precursor was retained within the carbon matrix (3–5 at.%). Figure 2d–f show the mapping of EDX of metal NPs on N-doped carbon. Based on the SEM and EDX images, the metal NPs are highly dispersed and quite uniformly distributed on N-doped carbon support.

**Figure 3:**
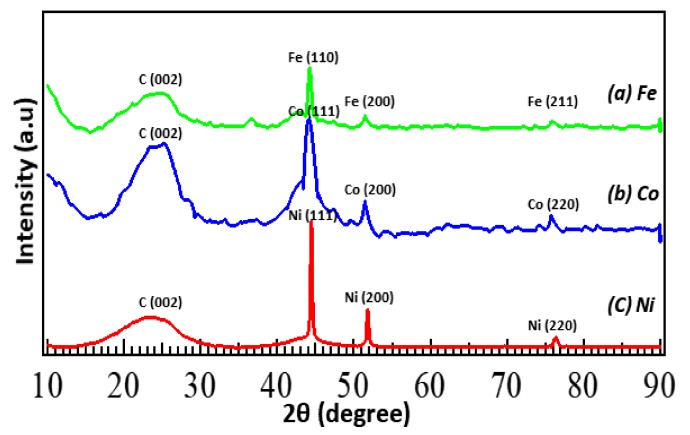
XRD patterns obtained for metal (Fe, Co, Ni)-N/C.

Table 2 summarizes the surface area and the average pore diameter of metal-N-doped carbons as estimated by the BET adsorption–desorption method. The surface area of metal-N-doped carbons is approximately 140 m^2^/g and the average pore diameter is 12–15 nm. Fe-N/C shows the largest surface area (145.3 m^2^/g) and average pore diameter (15.3 nm), followed by Co-N/C (144.8 m^2^/g) and Ni-N/C (138.6 m^2^/g). The N_2_ adsorption–desorption isotherm of Fe-N/C (Figure S1a) demonstrates a typical IV-type isotherm. A narrow hysteresis loop at higher relative pressures reveals that the N-doped carbon consists of mostly large size mesopores and interconnecting channels. From Figure S1b, the Barret–Joyner–Halenda (BJH) adsorption pore size distribution also confirms that the catalysts consist of mesoporous pore structure with 10–20 nm. Figure 3 shows the XRD patterns of the three metal (Fe, Co, Ni)-N-doped carbons. All three samples exhibit a similar pattern, with a broad peak of graphite (002) at 23.4°. Each metal (Fe, Co, Ni) peak at 44–45° (Fe [110], Co, Ni [111]), 51–52° (Fe, Co, Ni [200]) and 75–76° (Fe [211], Co, Ni [220]) indicates that the metal particles generated by electrode sputtering were successfully doped and present as its pure metal form on the N-doped carbon matrix.

### 3.2. Chemical Bonding States of Metal (Fe, Co, Ni)-N/C Catalyst

**Figure 4:**
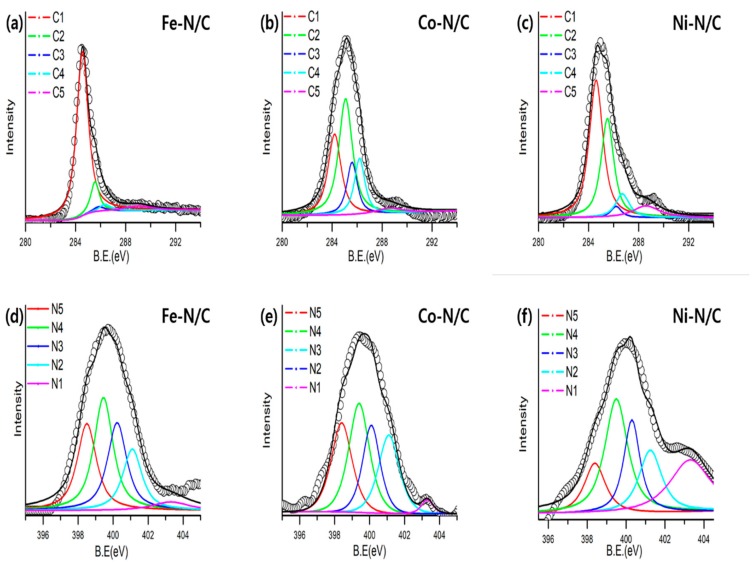
(**a**–**c**) High-resolution C 1s XPS spectra with peak deconvolution of metal (Fe, Co, Ni)-N/C, and (**d**–**f**) N 1s XPS spectra with peak deconvolution of metal (Fe, Co, Ni)-N/C.

Surface analyses were performed in order to analyze the chemical bonding type of N-doped carbon. The C1s spectra of each catalyst are shown in Figure 4a–c. All samples showed similar bonding states in the C1s spectra. The sp2 graphitic structure and sp3 C–C bonding (C1: 284.5 eV, C2: 285.6 eV) were the most dominant peaks, followed by C–N (C3: 286.0 eV), C–O (C4: 286.5 eV) and O–C=O (C-5: 288.7 eV) bonding. The presence of C–O and O–C=O are commonly due to the oxidation of the carbon surface [45]. Figure 4d–f demonstrate the narrow scan of N1s, which are further deconvoluted into 5 nitrogen-carbon (N-C) bonding states (N1: Oxide-N, N2: Pyrrolic-N, N3: Graphitic-N, N4: Amino-N, N5: Pyridinic-N). The amino-N (23–31%) clearly dominates in all metal-N/C catalyst, followed by graphitic-N (19–25%) and pyridinic-N (18–24%). The N–C bonding states are similar for all catalysts, which indicates that the structure of the aniline precursor affects the preference of the N–C bonding state. This result, therefore, confirms that the N–C bonding state of the catalyst synthesized by the solution plasma method is highly affected by the original precursor. Table 3 summarizes the N–C bonding types and their corresponding percentages of each catalyst.

### 3.3. Electrochemical Properties of the Metal (Fe, Co, Ni)-N/C Catalyst

**Figure 5:**
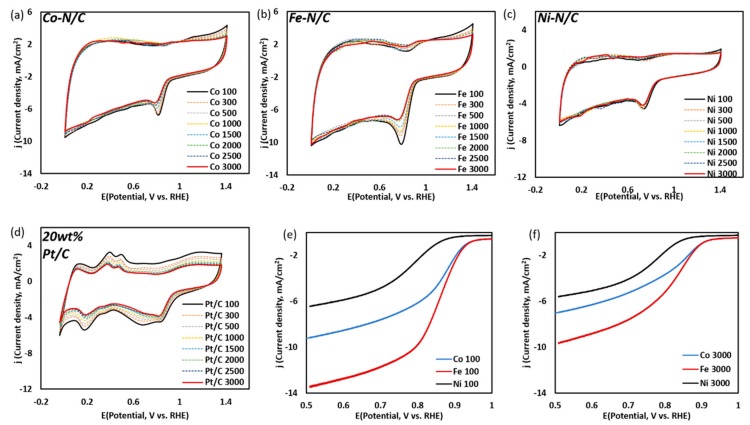
(**a**–**d**) CV curves of metal (Co, Fe, Ni)-N/C and 20 wt.% Pt/C from 100 to 3000 cycles at 50 mVs^−1^ in O_2_ saturated 0.1 M KOH: (**a**) Co-N/C, (**b**) Fe-N/C, (**c**) Ni-/C and (**d**) 20 wt.% Pt/C, and (**e**,**f**) LSV curves of metal (Co, Fe, Ni)-N/C at 10 mVs^−1^ scan rate in O_2_ saturated 0.1 M KOH electrolyte: (**e**) after 100 cycles, and (**f**) after 3000 cycles.

Figure 5a–d show CV polarization curves for metal-N/C and 20 wt.% Pt/C up to 3000 cycles at a scan rate of 50 mVs^−1^ in O_2_-saturated 0.1 M KOH solution. The ORR onset potential of Fe-N/C and Co-N/C are almost the same (0.93 V vs. RHE), where Ni-N/C exhibits a lower onset potential (0.88 V vs. RHE). Similar to other studies reported on transition metal-doped carbon, the catalytic activities of Fe-N/C and Co-N/C are normally higher than that of Ni-N/C [32]. Our group has already conducted metal-free amino-N-doped carbon with the plasma method and applied aniline as a C–N precursor. From the previous study, selective bonding was achieved with amino-N as the most pronounced bonding (49%), where its corresponding ORR onset potential was reported as 0.82 V vs. RHE [37]. Compared to that of the metal-free amino-N/C, current research suggests that metal NPs coordinated with a N-doped carbon matrix provide a synergic effect on advanced ORR performance.

The ORR ***E*_1/2_** potential of Fe-N/C and Co-N/C also show similarity (0.84 V vs. RHE (Co-N/C), 0.82 V vs. RHE (Fe-N/C)), where Ni-N/C exhibits a lower *E*_1/2_ potential (0.78 V vs. RHE). In contrast, Fe-N/C shows the highest *E*_1/2_ current density (5.12 mA/cm^2^), followed by Co-N/C (3.36 mA/cm^2^), and Ni-N/C (2.26 mA/cm^2^). Table 4 summarizes the onset potential and maximum current density at 100 and 3000 cycles, respectively, for each electrocatalyst. Although the current densities of all three as-synthesized catalysts decrease at 3000 cycles, their onset potentials remain relatively stable compared to that of 20 wt.% Pt/C. Table 4 summarizes the onset potential and maximum current density at 100 and 3000 cycles, respectively, for each electrocatalyst. Other studies have proposed that metal cations coordinated by graphitic-N and pyridinic-N play an important role in ORR activity [27,28,29,30]. Based on our result, we suggest that transition metal doped with selective amino-N bonding also has a positive synergetic effect on ORR activity.

**Figure 6:**
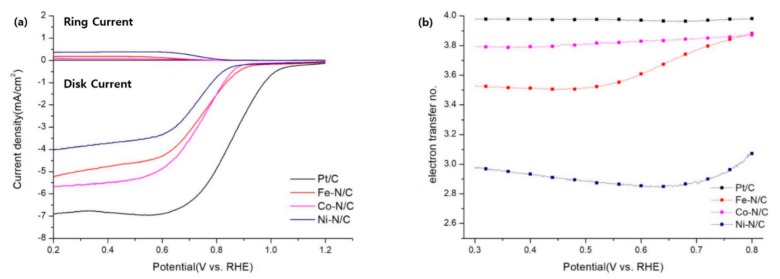
(**a**) RRDE analysis @1600 rpm of (Co, Fe, Ni)-N/C and 20 wt.% Pt/C at 10 mVs^−1^ in O_2_ saturated 0.1 M KOH solution at a scan rate of 10 mV s^−1^ and a rotation speed of 1600 rpm, and (**b**) the correlated electron number of (Co, Fe, Ni)-N/C and 20 wt.% Pt/C between 0.3 V to 0.8 V vs. RHE.

In Figure 6a,b, the electron transfer number (*n*) is derived from the equation n = $4\times \frac{id}{id+ir/Nc}$, where *i_d_* is the disk current, *i_r_* is the ring current, and *N_c_* is the collection efficiency (0.42 from the manufacturer). The behavior of the catalysts towards the ORR in alkaline media are as follows [46]:

When the overall reaction is 4-electron:

O_2_ + H_2_O + 4e^−^ → 4OH^−^ (1)

When it is a 2-electron reaction:

O_2_ + H_2_O + 2e^−^ → HO_2_^−^ (2)

followed by

HO_2_^−^ + H_2_O + 2e^−^ → 3OH^−^ (3)

The calculated electron transfer number (*n*) at 0.5 V vs RHE are in the order of 20 wt.%Pt/C (3.97) > Co-N/C (3.81) > Fe-N/C (3.50) > Ni-N/C (2.88). Normally, the ORR takes place mainly through a 2-electron pathway through HO_2_^−^ formation at the pristine carbon surfaces, whereas it follows a 4-electron pathway in Pt catalysts. As a result, the catalytic system of commercial 20 wt.% Pt/C is expected to find a mixed behavior between the two proposed mechanisms, but the electron transfer number *n* should be very close to 4 [46]. Among all transitional metal-N/C catalysts, Co-N/C shows the highest electron transfer number *n*, as well as a similar behavior to Pt/C. On the contrary, Fe-N/C and Ni-N/C exhibit a different trend compared to Co-N/C and Pt/C. The behavior of these two catalysts is almost the same compared to other N-doped carbon catalysts, of which their reported *n* values were 2.5–3.7 [47]. As the major C–N bondings of all transitional metal-N/C catalysts have no major differences, it is easy to observe that Co NP provides higher selectivity of the 4-electron pathway.

## 4. Conclusions

Transitional metal/amino-N-doped carbon was synthesized via a plasma process using aniline and a transition metals (Fe, Co, Ni) electrode as starting precursors. Both SEM and XRD analyses confirmed that the metal NPs were successfully doped on the N-doped carbon support. Surface analysis revealed that amino-N was the dominant bonding state in all electrocatalysts, which suggests that the starting precursor played a significant role in the chemical bonding state. Compared to metal-free N-doped carbon, metal NPs on N-doped carbon have a synergic effect on advanced ORR performance. In the electrochemical testing, Co-N/C carbon showed the best ***E*_1/2_** potential (0.84 V vs. RHE) and Fe-N/C exhibited the highest ***E*_1/2_** current density (5.12 mA/cm^2^). Within all types of TM-NPs-N/C catalyst, Co-N/C showed the highest onset ORR potential and high selectivity in the 4e^−^ reaction pathway. Although its catalytic performance was still slightly lower than that of commercial 20 wt.% Pt catalyst, its low cost, and relatively high catalytic performance hold promise as alternative ORR electrocatalysts in the practical metal-air battery.

## Figures and Tables

**Table 1:** Atomic percentages of C, N, H of each metal (Fe, Co, Ni)-N/C from EDS.

	Co-N/C	Fe-N/C	Ni-N/C
**C (at. %)**	83.4%	85.5%	79.8%
**N (at. %)**	5.1%	5.6%	3.2%
**H (at. %)**	2.4%	1.51%	1.1%

**Table 2:** Textural parameters of metal (Co, Fe, Ni)-N/C derived from the N_2_ adsorption–desorption isotherms.

	Co-N/C	Fe-N/C	Ni-N/C
**BET surface area**	144.8 m^2^/g	145.3 m^2^/g	138.6 m^2^/g
**BJH Adsorption pore average width**	12 nm	15.3 nm	12.8 nm

**Table 3:** Bonding states of nitrogen-carbon in metal (Co, Fe, Ni)-N-doped catalysts from deconvolution of N 1s spectra.

Bonding	Binding Energy	Co-N/C	Fe-N/C	Ni-N/C
		Relative Percentage (%)		
**N1(Oxide-N)**	403.3–403.7 eV	5	4.3	16.2
**N2(Pyrrolic-N)**	401.1–401.5 eV	20.6	16.8	16.5
**N3(Graphitic-N)**	400.1–400.3 eV	21.1	24.1	21.2
**N4(Amino-N)**	399.4–399.6 eV	28.7	31	26.7
**N5(Pyridinic-N)**	398.4–398.6 eV	24.6	23.8	19.4

**Table 4:** Summary of the electrochemical catalytic activity of metal (Co, Fe, Ni)-N/C and 20 wt.% Pt/C.

	Co-N/C	Fe-N/C	Ni-N/C	20 wt.% Pt/C
**Onset Potential** **@100 cycles**	0.93 V vs. RHE	0.93 V vs. RHE	0.88 V vs. RHE	1.03 V vs. RHE
**Potential E_1/2_** **@100 cycles**	0.84 V vs. RHE	0.82 V vs. RHE	0.78 V vs. RHE	0.94 V vs. RHE
**Current density at E_1/2_ @100 cycles**	3.36 mA/cm^2^	5.12 mA/cm^2^	2.26 mA/cm^2^	2.22 mA/cm^2^
**Onset Potential** **@3000 cycles**	0.90 V vs. RHE	0.90 V vs. RHE	0.85 V vs. RHE	0.94 V vs. RHE
**Potential E_1/2_** **@3000 cycles**	0.83 V vs. RHE	0.81 V vs. RHE	0.77 V vs. RHE	0.91 V vs. RHE
**Current density at E_1/2_ @3000 cycles**	2.07 mA/cm^2^	3.62 mA/cm^2^	2.07 mA/cm^2^	1.90 mA/cm^2^

## Supplementary Materials

The following are available online at https://www.mdpi.com/2079-4991/9/5/742/s1. BET adsorption -desorption of Fe-N/C, (a) Isotherm linear adsorption-desorption plot of Fe-N/C, (b) BJH adsorption pore size distribution.
