# Supplementary material for: Transition Metal (Fe, Co, Ni) Nanoparticles on Selective Amino-N-Doped Carbon as High-Performance Oxygen Reduction Reaction Electrocatalyst
Source: Nanomaterials (Basel). 2019 May 14;9(5):742. doi: 10.3390/nano9050742 (PMC6566341; doi:10.3390/nano9050742)
Supplement: Supplementary file 1 [file nanomaterials-09-00742-s001.pdf]

Supplementary data:

# Transition Metal (Fe, Co, Ni) Nanoparticles on Selective Amino-N-Doped Carbon as High-Performance Oxygen Reduction Reaction Electrocatalyst

**Seonghee Kim [1], Shuhei Kato [2], Takahiro Ishizaki [2], Oi Lun Li [1,*] and Jun Kang [3,*]**

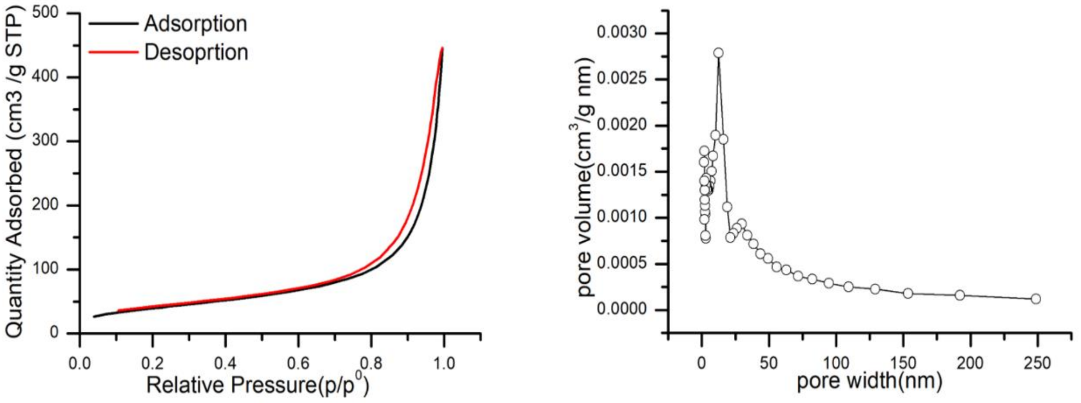


**Figure S1.** BET adsorption-desorption of Fe-N/C, (**a**) Isotherm linear adsorption-desorption plot of Fe-N/C, (**b**) BJH adsorption pore size distribution.
